# A qPCR-Based Method for Quantification of RCA Contaminants in Oncolytic Adenovirus Products

**DOI:** 10.3389/fmolb.2022.883249

**Published:** 2022-05-23

**Authors:** Menghan Gao, Erik Yngve, Di Yu, Chuan Jin

**Affiliations:** Department of Immunology, Genetics and Pathology, Science for Life Laboratory, Uppsala University, Uppsala, Sweden

**Keywords:** replication-competent adenovirus, conditionally replicating adenovirus, quantification, clinical production, qPCR, RCA contaminants

## Abstract

Oncolytic adenovirus is one of the most promising treatments against cancer and is widely evaluated clinically. During high titer production, “Wild-type-” like replication-competent adenovirus (RCA) contaminants can be generated through recombination events due to the DNA sequence similarity between oncolytic virus and host cells. These RCA contaminants raise various safety concerns in clinics. Cell culture-based methods have been developed to detect RCA contaminants in replication-deficient adenovirus vectors. These methods were based on that only RCA contaminants, but not the vectors, are able to grow in and lyse the test cell line. However, these methods are not suitable for distinguishing RCA contaminants from the oncolytic adenovirus products because both can replicate in test cell lines. Herein, we reported a qPCR-based method to quantify RCA contaminants quickly and reliably in E1B-deleted oncolytic adenovirus products. This method is based on specific detection of the E1B gene, which can be acquired during production *via* recombination events between viral and host cell DNA. The assay is sensitive with the limit of detection at 10 VP of the RCA contaminants and the limit of quantification at 75 VP of the RCA contaminants in each 40 µL qPCR reaction. We have also validated the method on virus batches produced in the non-GMP and GMP conditions. Our results showed that this qPCR-based method was reliable and robust for detecting and quantifying RCA contaminants in oncolytic adenovirus products. The method may also be adapted for other oncolytic adenoviruses products by switching primer sets.

## Introduction

Oncolytic adenoviruses (OAdVs) are advanced medical products with increasing applications within cancer therapy. OAdVs are genetically engineered to replicate in tumor cells but not in normal cells (Everts and Van Der Poel, 2005). OAdVs are usually either constructed with tumor-/tissue-specific promoters controlling E1 expression (e.g., AdVince) (Yu et al., 2017) or through deletion of some part of the E1 gene (e.g., DNX-2401 and ONYX-15) (Wold and Toth, 2013; Heise et al., 1997). In contrast to oncolytic adenovirus, recombinant adenoviral vectors are often replication-incompetent, and their essential viral E1 sequence is usually replaced by therapeutic genes (Yu et al., 2017). They are also widely used in clinical gene therapy (Yu et al., 2017).

Many adenoviral products, including oncolytic adenovirus and recombinant adenovirus vectors, are produced in HEK293 cells. The adenoviral genome 1-4344 (Louis et al., 1997) presented in this cell line provides the complementary E1-function for E1-deleted adenovirus. Similarly, another adenovirus-producing cell line 911 contains the 79-5789 adenoviral genome (Fallaux et al., 1996) to compensate for E1 function. Therefore, the presence of the E1 gene in producer cells may cause undesirable generation of “wild-type-” like replication-competent adenovirus (RCA) contaminants through recombination between viral and host cell DNA (Lochmüller et al., 1994). Herein, we use the term RCA to strictly refer to the RCA contaminants. These RCA contaminants constitute a risk of unintended viral spread and host inflammation response when the viral products are used clinically (U.S. Department of Health and Human Services et al., 2001). RCA contaminants can be avoided using cell lines containing a minimized adenoviral sequence (e.g., Per.C6 with adenoviral genome 459-3510) (Fallaux et al., 1998) or completely lacking the E1 gene (e.g., A549) to abolish RCA contaminants formation. Note that E1-free cell lines such as A549 can only be utilized for oncolytic adenoviruses, which retain replication capacity in cancer cells. However, to warrant safety, it is crucial to determine the number of RCA contaminants in each batch of oncolytic adenoviruses intended for clinical use (Fallaux et al., 1999). Replication-deficient adenoviral vectors can reliably be tested for RCA contaminants through cell-based assays, such as the cell culture/cytopathic effect (CPE) assay (Zhu et al., 1999), based on the out-growth of RCA contaminants. However, such methods cannot distinguish RCA contaminants from the actual oncolytic virus due to the lack of cell lines that only support the growth of RCA contaminants. Therefore, it is emerging to develop an accurate, sensitive, and robust method to examine the level of RCA contaminants in clinical batches of oncolytic adenovirus products.

In this study, we designed a real-time quantitative PCR (qPCR)-based assay that can be used to detect and quantify RCA contaminants in batches of E1B-deleted oncolytic adenoviruses. Recombination between the viral and the producer cell line genomes will produce a recombinant DNA template that can be detected. The assay utilizes a primer set that specifically binds to the E1B-region absent in the oncolytic virus but present in the producer cell genome and RCA contaminants. Thus, only the RCA contaminants are detected in the purified product. Our assay has a low limit of detection (LLOD) of 10 VP and a low limit of quantification (LLOQ) (95% confidence) of 75 VP in each 40 µL qPCR, presenting a high sensitivity and accuracy for monitoring potential RCA contaminants in clinical products. In addition, using this method, we also evaluate potential RCA contaminants for our GMP products intended for clinical usage.

## Materials and Equipment

• Cell lines for virus production: human embryonic retinoblasts cell line 911 (a kind gift from Crucell, Netherlands) and human lung carcinoma epithelial cell line A549 (purchased from ATCC).
• Wild-type adenovirus 5 (Ad5wt) (purchased from ATCC).
• E1B-deleted oncolytic adenovirus virus (Ad5dE1B) derived from 911 (Ad5dE1B_911) or A549 (Ad5dE1B_A549) cell line.
• Oncolytic adenovirus produced under GMP condition (Ad5dE1B_GMP).
• 10 mM Tris-HCl: prepared by 1 m Tris-HCl, pH 8.0 (Invitrogen, AM9855G), and nuclease-free water (Invitrogen, AM9939).
• Lysis buffer: prepared by Proteinase K (Thermo Scientific, EO0491) and 10 mM Tris-HCl.
• Primer sets for RCA contaminants quantification: Fwd-AdE1B55K 5′- GCC​GAG​GTG​GAG​ATA​GAT​A-3′ and Rev-AdE1B55K 5′-CGT​GTA​GGA​TAA​GGT​TGG​TAT​T-3′ (target region 2072-2240); Fwd-AdE1B19K 5′-TTC​TGC​TGT​GCG​TAA​CTT​G-3′ and Rev-AdE1B19K 5′-TCT​TGA​TGA​CCT​TCT​CTT​GGA-3′ (target region 1202-1392).
• SYBR Green PCR Master Mix (Applied Biosystems, 4309155).
• Thermocycler with fluorophore detector for qPCR (BioRad CFX96 system).

## Methods

The method is generally based on qPCR detection of RCA contaminants in E1B-deleted oncolytic adenoviruses. To minimize DNA loss and maximize detection of RCA contaminants, viral DNA was released using proteinase digestion of viral capsid without further purification steps. The digested product was directly subjected to qPCR analysis. Serial dilutions of wild-type Ad5 (Ad5wt) were used to generate the standard curve for quantification. A detailed protocol is described in the following.

### Cell Culture

Human embryonic retinoblasts cell line 911 (Crucell, Netherlands) was maintained in DMEM (Gibco) with 10% fetal bovine serum (FBS) (Gibco), 100 U/ml penicillin-streptomycin (PEST) (Gibco), and 1 mM sodium pyruvate (NaPyr) (Gibco). Human lung carcinoma epithelial cell line A549 (ATCC) was maintained in RPMI-1640 (Gibco) supplemented with 10% FBS (Gibco), 100 U/ml PEST (Gibco) and 1 mM NaPyr (Gibco). All cells were cultured in a humidified incubator at 37°C with 5% CO_2_.

### Production and Titration of Adenovirus

Wild-type (WT) adenovirus, designated as Ad5wt, was propagated in A549 cells. The genetically engineered oncolytic adenovirus Ad5dE1B was produced in 911 or A549 cell lines, designated as Ad5dE1B_911 and Ad5dE1B_A549. These viruses were produced in non-GMP conditions and purified by CsCl density-gradient centrifugation (Yu et al., 2011). Ad5dE1B was also produced in GMP condition using the A549 producer cell line and designated Ad5dE1B_GMP. Virus titer (viral particles, VP) (Table 1) was determined by measuring absorbance at 260 nm as described (Maizel et al., 1968).

**TABLE 1:** Titer (VP) of different batches of viruses used in the study.

Virus batch	Producer cell line	Titer (OD_260_) (VP/µL)	Production condition
Ad5wt	A549	6.6×10^9^	Lab batch
Ad5dE1B_A549	A549	8.5×10^9^	Lab batch
Ad5dE1B_911	911	6.7×10^9^	Lab batch
Ad5dE1B_GMP	A549	5.17×10^9^	GMP

### Real-Time Quantitative PCR

Lysis buffer was prepared by diluting Proteinase K in 10 mM Tris-HCl to a final concentration of 1 mg/ml (>30 U/ml). Here, proteinase K is overloaded to achieve maximum release of viral DNA. The concentration of proteinase K could be titrated down to fit the optimal condition. The serially diluted Ad5wt (for standard curve) or undiluted test samples were directly added to lysis buffer (total 18 µL containing 2 μL sample plus 16 μL lysis buffer) and incubated at 37°C for 16 h to release viral genomic DNA. Each sample was then heated at 100°C for 10 min to inactivate the proteinase. Primers (1 μL of each with a final concentration at 5 µM) and 20 µL of 2× SYBR Green PCR Master Mix were added to each sample (a total of 40 μL per reaction). qPCR was performed using cycling conditions: denaturation at 95°C for 5 min, followed by 45 cycles of denaturation at 95°C for 30 s, annealing at 55°C for 30 s, and extension at 72°C for 1 min. The signal was read at the end of each cycle. The melting curve was generated by increasing the temperature from 65°C to 95°C with an increment of 0.5°C per 5 s.

### Statistical Analyses

For further analysis, a linear regression curve was fitted to the Ct values and the log-transformed virus particle quantity. As the no template control (blank water sample) gives no detectable signal, the lower limit of detection (LLOD) was set to the lowest dilution point tested in the assay. The lower limit of quantification (LLOQ) was then calculated by the formula LLOQ = LLOD + 3.2 × SD_*LLOD*_.

## Results

### Determine the Low Limit of Detection and Low Limit of Quantification

**FIGURE 1:**
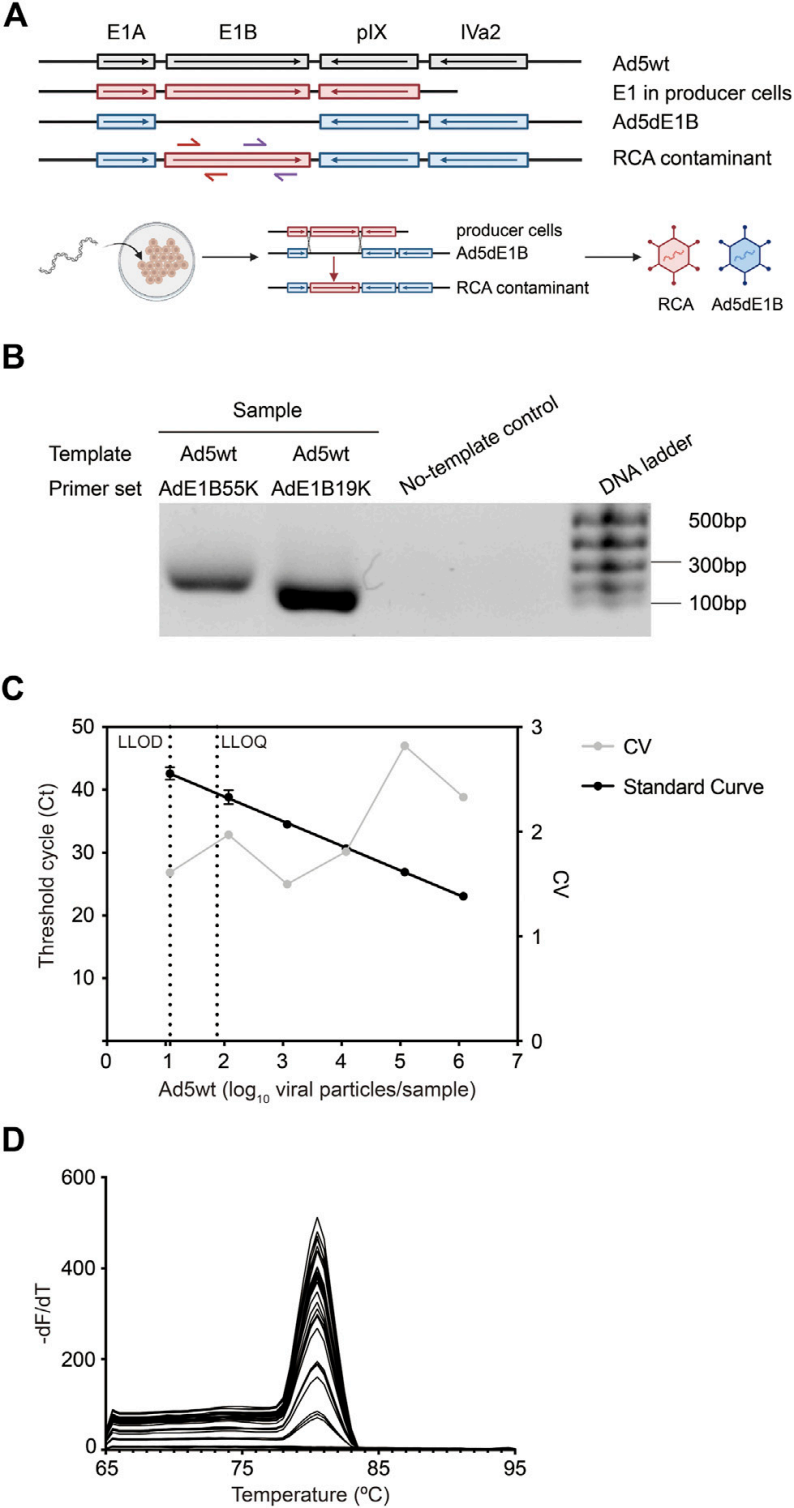
Sensitivity and accuracy of the established qPCR. **(A)** Schematic overview of the 5′-end of adenoviral DNA templates. PCR primer sets Fwd-AdE1B19K/Rev-AdE1B19K (red arrows) and Fwd-AdE1B55K/Rev-AdE1B55K (purple arrows) are indicated at RCA contaminants. An illustration of the generation of RCA contaminants through recombination is shown in the lower panel. Ad5wt, wild-type adenovirus 5; Ad5dE1B, E1B-deleted adenovirus; RCA, replication-competent adenovirus. **(B)** Verification of the primer specificity by gel electrophoresis. Primer sets Fwd-AdE1B19K/Rev-AdE1B19K and Fwd-AdE1B55K/Rev-AdE1B55K were used to amplify Ad5wt and the amplicons were resolved in DNA gel electrophoresis. **(C)** Representative standard curve for the detection assay, showing the mean Ct value ± SD (*n* = 6) (left Y-axis) against log_10_-transformed VP of Ad5wt. The inter-assay coefficients of variability (CV, right Y-axis) are shown in gray. The average PCR efficiency of different repeats is 90.9 ± 4.8%. LLOD, the lower limit of detection. LLOQ, the lower limit of quantification. **(D)** Amplicon melting curves of final qPCR products generated using primer set Fwd-AdE1B19K/Rev-AdE1B19K in serial diluted Ad5wt samples.

Two primer sets, Fwd-AdE1B55K/Rev-AdE1B55K and Fwd-AdE1B19K/Rev-AdE1B19K, were designed targeting either E1B 55K or 19K region as indicated in Figure 1A. Both primer sets target an absent region in our E1B-deleted oncolytic virus Ad5dE1B but will be present in the RCA contaminants. When evaluated by standard PCR, non-specific amplification was observed in neither of the two primer pairs (Figure 1B), verifying the specificity of these primer pairs. We thus selected Fwd-AdE1B19K/Rev-AdE1B19K to continue the method development. Ad5wt was serially diluted in 10 mM Tris-HCl and used to mimic RCA contaminants in the following qPCR assay. We plotted the Ct values against the log-transformed virus particle quantity to obtain a well linearized standard curve when analyzing serially diluted samples containing wild-type adenovirus (Figure 1C). The goodness-of-fit is usually R^2^ > 0.98, indicating the robustness of the assay. Because no amplification signal was detected in the negative sample, we set the lower limit of detection (LLOD) at the lowest dilution point tested in this assay (10 VP per 40 µL qPCR), which gives the lower limit of quantification (LLOQ) at 75 VP per 40 µL qPCR with 95% confidence (Figure 1C). Additionally, a single peak in the melting curve also verified the specificity of the primer set Fwd-AdE1B19K/Rev-AdE1B19K (Figure 1D).

### Detection of Replication-Competent Adenovirus Contaminants Present in Lab-Batch E1B-Deleted Oncolytic Adenovirus

**FIGURE 2:**
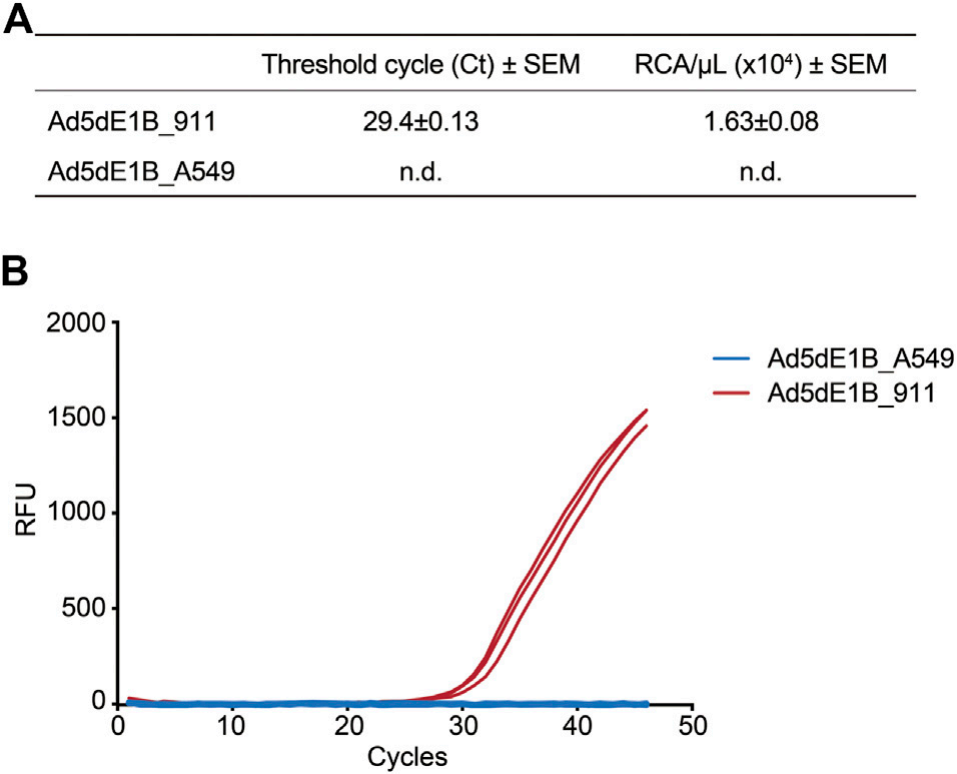
qPCR-based quantification of RCA contaminants in lab batches of E1B-deleted oncolytic adenovirus. **(A)** Ct values and RCA contaminants of different batches of virus produced in A549 and 911 cells (*n* = 3). n.d., not detectable. **(B)** Representative amplification curves of two batches of Ad5dE1B produced in either 911 (red) or A549 (blue).

Next, we detected and quantified the RCA contaminants in E1B-deleted oncolytic adenovirus virus (Ad5dE1B) produced in either 911 (Ad5dE1B_911) or A549 (Ad5dE1B_A549) cells. Ad5dE1B_A549 did not show any amplification signal after 45 cycles, indicating the RCA contaminants were below our detection limitation (Figures 2A,B). In clear contrast, Ad5dE1B_911 showed a Ct-value around 30, indicating 1.630 × 10^4^ VP of RCA contaminants in 1.34 × 10^10^ VP (in 2 µL tested sample) of the virus produced in the 911 cell line (Figure 2A).

### Detection of Ad5wt (Mimetic of Replication-Competent Adenovirus Contaminants) Spiked in Different Batches of E1B-Deleted Oncolytic Adenovirus Shows the Robustness of the Method

**FIGURE 3:**
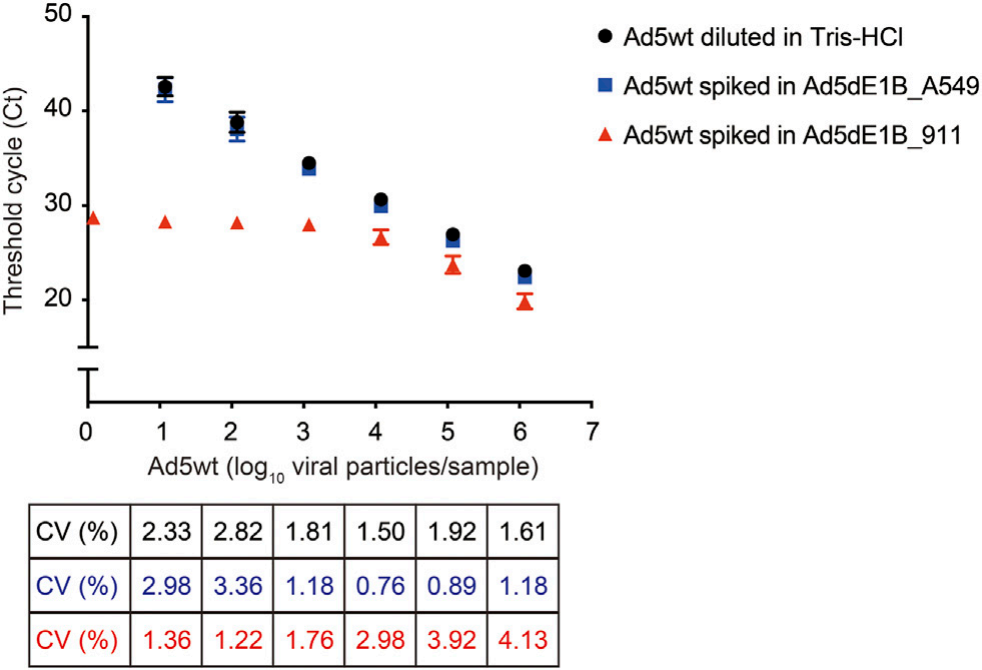
qPCR quantification of replication-competent Ad5 spiked in E1B-deleted adenovirus produced in 911 or A549 cells. Different amounts of Ad5wt (as RCA contaminants mimetic) were spiked into E1B-deleted adenovirus produced in A549 or 911. Threshold cycle (Ct) was determined by qPCR (*n* = 6 for Ad5wt diluted in 10 mM Tris-HCl and spiked in Ad5dE1B_A549 samples; *n* = 2 for Ad5wt spiked in Ad5dE1B_911 samples; N indicates biological experiment repeats with triplicates samples in each experiment). The inter-assay coefficients of variability (CV) are indicated below.

To test the robustness of the assay and evaluate whether background adenoviral genome DNA can affect the sensitivity, different amounts of Ad5wt (10^0^, 10^1^, 10^2^, 10^3^, 10^4^, 10^5^, 10^6^), serving as mimetic of RCA contaminants, were spiked into either 8.5 × 10^9^ VP/µL of Ad5dE1B_A549 or 6.7 × 10^9^ VP/µL of Ad5dE1B_911. Ad5wt spiked into Ad5dE1B_A549 showed a similar Ct value as Ad5wt diluted in 10 mm Tris-HCl, indicating the existence of excessive other adenoviral DNA sequences does not interfere with the detection (Figure 3). This warrants primer specificity. On the contrary, in the Ad5dE1B_911 spiked sample, we observed stabilization of the Ct values when the spiked Ad5wt was below 10^4^ VP (Figure 3), further confirming the presence of RCA contaminants in the virus batch produced in 911 cells (Figures 2A,B).

### Detection of Replication-Competent Adenovirus Contaminants Present in a GMP-Grade Batch of E1B Deletion Oncolytic Adenovirus

**FIGURE 4:**
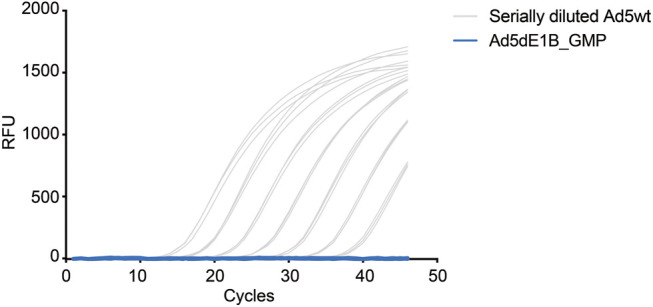
qPCR-based quantification of RCA contaminants in GMP-grade E1B-deleted oncolytic adenovirus. Representative amplification curves of serially diluted Ad5wt in 10 mM Tris-HCl for the standard curve (gray) and a batch of GMP-grade E1B-deleted oncolytic adenovirus (blue) produced in A549 cells (5.17 × 10^9^ VP/µL). The experiments were performed independently by two operators with three repeats on different days. Triplicate samples were used in each test.

Based on the previous evaluation, the method was proven accurate and reliable in detecting RCA contaminants in highly concentrated adenoviral products. Next, we use this assay to evaluate the RCA contaminant level of an E1B-deleted adenovirus (Ad5dE1B_GMP) produced in A549 cells at the GMP facility at Baylor College of Medicine. Serially diluted Ad5wt virus was used as standard (Figure 4). As expected, no amplification signal could be detected in the clinical GMP-batch of the virus (Figure 4), confirming less than 10 VP of RCA contaminants in the oncolytic virus produced in A549 cells in 2 µL tested sample (equivalent to 1.03 VP × 10^10^ VP).

## Discussion

Oncolytic adenoviruses are currently developed to fight cancer due to their tumor selectivity, safety, and capability to deliver transgenes and stimulate immune responses against tumor cells (Tripodi et al., 2021). Several clinical trials evaluate oncolytic adenovirus as either a single therapy or in combination with conventional cancer therapies and immune checkpoint inhibitors (Mondal et al., 2020).

Currently, HEK293 is still the primary producer cell line for producing both recombinant adenoviral vectors and oncolytic adenoviruses. Due to the presence of E1 gene DNA in its genome, HEK293 cells are prone to generate RCA contaminants during production. Thus, quantification of the presence of RCA contaminants is critical for warranting the safety of clinical viral products. Assays based on cell culture and cytopathic effect (CPE) after viral infection have generally been used for RCA contaminants quantification for adenoviral vector products. The presence of RCA contaminants is judged manually by microscopic observation, and thus the results may not always be accurate and quantitative (Marzio et al., 2007). Moreover, researchers also showed that the cell-culture-based method could be combined with qPCR to improve detection sensitivity (Ishii-Watabe et al., 2003; Schalk et al., 2007). However, the oncolytic adenovirus presents an additional challenge as its proliferation capacity is retained and cannot be distinguished using a cell-culture-based CPE assay. Therefore, it is emerging to develop an accurate and sensitive assay to detect and quantify RCA contaminants for oncolytic adenoviruses.

Since E1B-deleted oncolytic adenoviruses are evaluated in several clinical trials (Habib et al., 2002; Mulvihill et al., 2001; Lu et al., 2004) (Mulvihill et al., 2001; Habib et al., 2002; Lu et al., 2004), our qPCR-based assay takes advantage of the sequence difference between the actual virus product and RCA contaminants. This difference allows us to design premiers targeting E1B specifically (Figures 1A,B) and thus distinguish between RCA contaminants and the E1B-deleted oncolytic virus, which is fundamental in the assay design. Based on the sensitivity of PCR, we achieved LLOD of 10 VP of RCA contaminants presented in each reaction.

When developing an assay for RCA contaminants quantification, one challenge is detecting a very low number of RCA contaminants among high concentrated viral particles. Therefore, we also validate our method using wild-type virus spiked samples to mimic this scenario, aiming to examine the specificity and sensitivity. Encouragingly, the regression curves show no difference between Ad5wt diluted in 10 mM Tris-HCl and Ad5wt spiked in Ad5dE1B_A549, indicating the method’s robustness and affirming that the method can be applied to clinical oncolytic adenoviral products. The signal detected in Ad5dE1B_911 further confirmed the presence of RCA contaminants in the virus batch produced in the 911 cell line.

Others have also reported methods for detecting and quantifying RCA contaminants in oncolytic viruses by performing differential amplification steps (Walker et al., 2003), wherein the test virus was passaged sequentially on a normal human fibroblast cell line. RCA contaminants are then determined by combinational assessment of the cytopathic effects, total viral productivity, increasing potency of killing normal cells, and restriction endonuclease digest analysis of aberrant vector genome structure. This method can be reliable and objective, but the whole procedure is quite complex and time-consuming, which might involve human errors. Our method is a one-step qPCR-based method, which is sensitive, accurate, and robust. Similar methods can be developed specifically for each different oncolytic virus product by switching the specific corresponding primer sets. The method can also be applied to the detection of RCA events in patients treated with E1B-deleted oncolytic virus as, in the case of infection, wild-type viral genome present in the patients could lead to the generation of RCA contaminants *via* recombination. In this case, primer sets should be optimized and designed to distinguish the RCA events from the wild-type viruses.

Conclusively, we report a time-saving qPCR-based method specifically for quantifying RCA contaminants from conditionally replicating oncolytic adenovirus, with high sensitivity and robustness.

## Data Availability

The original contributions presented in the study are included in the article/supplementary material. Further inquiries can be directed to the corresponding author.
